# Atypical Small Hemangiomas of the Liver: Hypervascular Hemangiomas

**Published:** 2007-12

**Authors:** Kais Nouira, Radhouane Allani, Iheb Bougamra, Khaled Bouzaidi, Olfa Azaiez, Habiba Mizouni, Monia Ben Messaoud, Emna Menif

**Affiliations:** *Department of Radiology, La Rabta Hospital, Tunis, Tunisia*

**Keywords:** hypervascular hemangioma, CT scan, MRI

## Abstract

Hyperdynamic hemangiomas (HH) are atypical hepatic hemangiomas with an incidence of approximately 16% of all hemangiomas in the liver. We report a case of HH in a 47-year-old woman. Multiphase helical CT scan, MRI appearances and differential diagnoses are discussed.

## INTRODUCTION

Most cavernous hemangiomas are easily distinguished from malignant hepatic tumors using multiphase computed tomography (CT) scan due to characteristic features. We report a case of atypical hemangiomas: hypervascular or hyperdynamic hemangiomas (HH) in which the appearance on helical enhanced CT scan is very particular and specific.

## CASE REPORT

**Figure 1:**
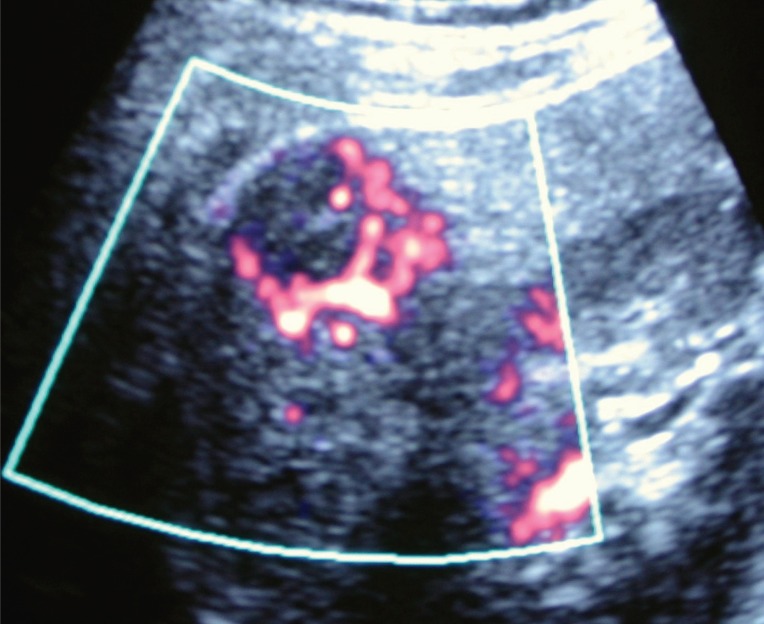
Power Doppler showing flow signals in and around the nodule of the segment 6 of the liver.

A 47-year-old woman with a history of stomach ulcer, presented with right hypochondriac pain. No abnormal findings were revealed by physical examination. Her liver function tests were within normal limits. Abdominal ultrasonography (US) revealed 3 nodules in segments 4, 6 and 7 measuring 10mm, 15mm and 20mm in diameter respectively. All nodules were well delineated and appeared hypoechoic with posterior acoustic shadow. Power Doppler showed vascular flow signals with arterial and venous flows on pulse Doppler in and around the nodule of the segment 6 (Figure 1).

**Figure 2:**
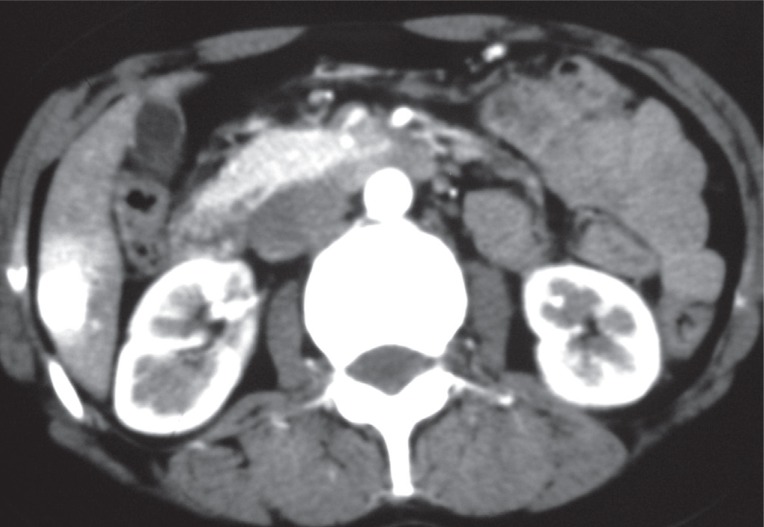
Axial CT scan after contrast injection in arterial phase. Hepatic hypervascular hemangioma with similar enhancement to the aorta and early enhancement of parenchyma adjacent to the lesion.

**Figure 3:**
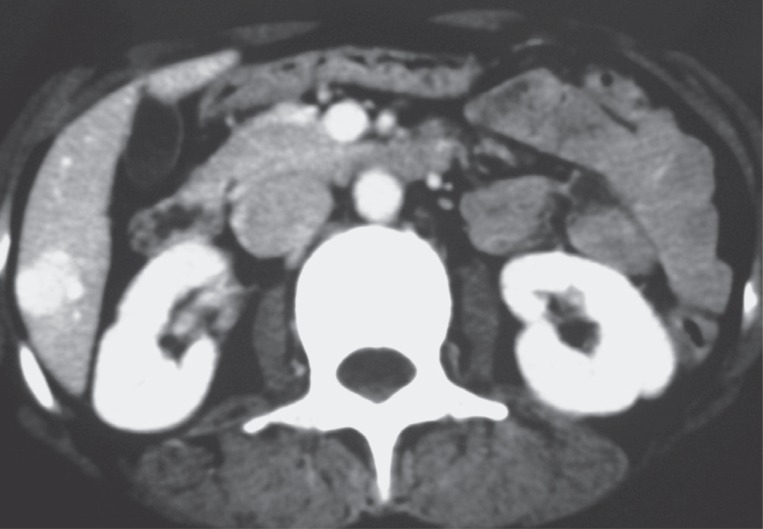
Axial CT scan after contrast injection in portal phase. The hepatic hemangioma has the same enhancement than that of the aorta.

Helical CT showed an early and complete homogeneous high enhancement of these nodules, which begins at the arterial phase and is parallel to the kinetic of enhancement of aorta in all the different phases of the examination and was accompanied by a wedge-shaped parenchymal enhancement adjacent to the tumors (Figures 2, 3).

**Figure 4:**
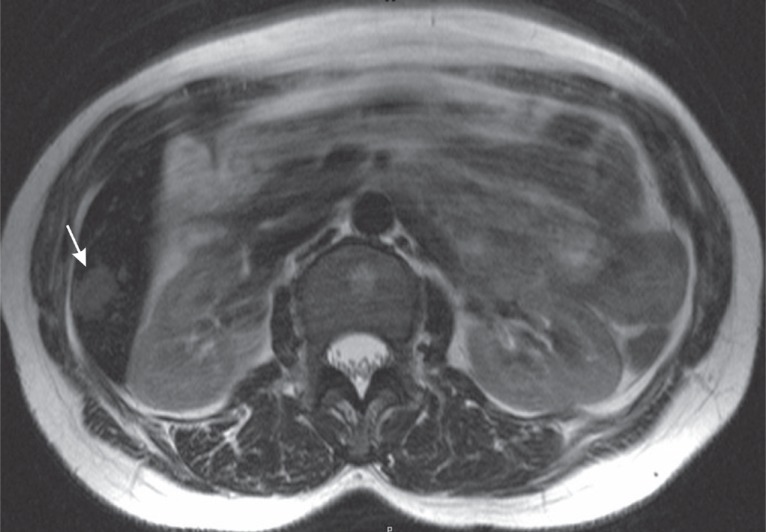
Axial T2 weighed MRI sequence. The hepatic hemangioma has an atypical T2 signal intensity (arrow) which is not as bright as cerebrospinal fluid as usually found in hepatic hemangiomas.

MRI showed that theses nodules appeared hyperintense with lower signal intensity compared to cerebrospinal fluid on T2 sequences (Figure 4). On T1 sequences they were hypointense and enhanced rapidly with the same dynamic enhancement as on helical CT.

The diagnosis of HH was made and regular follow up was indicated. After 2 years, these nodules showed the same size and enhancement on helical CT.

## DISCUSSION

Cavernous hemangiomas are the most common benign tumors of the liver. They can be found at any age and women are more likely than men to develop them. Hemangiomas are frequently in a subcapsular location, more commonly in the right lobe, especially the posterior segment. They are usually solitary but may be multiple (1, 2). They are usually asymptomatic and are, nowadays, diagnosed more often thanks to the important development of imaging.

Cavernous hemangiomas show typically, on Ct scans, a globular peripheral enhancement with slow progressive centripetal filling after bolus injection of contrast material. The complete isoattenuating fill-in occurs no less than three minutes and not more than 60 minutes after contrast material injection (3, 4). There is seldom doubt about the diagnosis of these tumors.

HH tend to be seen as hypoechoic lesions at sonography, as reported here. Color Doppler sonography may reveal intratumoral flows, large feeding arteries, and reversal of portal flow around the tumor. Knowledge of such sonographic findings may ensure an accurate sonographic diagnosis of these tumors (5, 6).

HH are atypical hepatic hemangiomas with an approximate incidence near to 16% of all angiomas. CT findings are usually characteristic and specific. They consist of an early, complete, and homogenous high enhancement which begins since the arterial phase and is parallel to the kinetic of enhancement of aorta in all the different phases of examination. This enhancement persists on late phase (1, 7, 8). In findings previously reported by Leslie et al, enhancement isointense to the aorta was 100% specific for HH (4).

At MRI, hepatic hemangiomas show typically bright signal intensity on T2 sequences which is one of the most reliable findings in the diagnosis. In HH, T2 signal intensity may be not as bright as cerebrospinal fluid on MRI, as in the current case, and may cause confusion. CT scan is thus diagnostically very helpful by showing enhancement similar to the aorta (7, 8, 9).

HH are often associated with an early enhancement of parenchyma adjacent to the lesion observed in arterial CT phase. This is considered to be mainly due to arterioportal shunts and portal obstruction or decreases in portal flow (5, 10). Arterioportal shunt associated with a hepatic tumor is generally recognized to be most characteristic of malignant tumors. However results of more recent studies have shown that arterioportal shunts are commonly seen in hepatic hemangiomas and especially in HH (5, 11).

Differential diagnosis can be made with other hypervascular hepatic lesions such as hypervascular metastasis, hepatocellular carcinomas, adenoma and focal nodular hyperplasia. Enhancement similar to that of the aorta in all phases is the main criteria to make differential diagnosis and avoid liver biopsy.

## CONCLUSION

HH show high, early and homogenous enhancement similar to that of aorta in all phases on helical Ct scan. It may be associated with arterioportal shunts and may have a variable T2 signal intensity on MRI. Multiphase helical CT scan makes a reliable diagnosis.
